# Outcome-Based Evaluations of Social Interaction Valence in a Contingent Response Context

**DOI:** 10.3389/fpsyg.2019.02557

**Published:** 2019-11-20

**Authors:** Jun Yin, Xiaoyan He, Yisong Yang, Xiaoying Wu

**Affiliations:** ^1^Department of Psychology, Ningbo University, Ningbo, China; ^2^Center of Group Behavior and Social Psychological Service, Ningbo University, Ningbo, China; ^3^Department of Psychology and Behavioral Sciences, Zhejiang University, Hangzhou, China

**Keywords:** social interaction, contingent interaction, intent, outcome, causality

## Abstract

Previous studies have indicated that social evaluations rely heavily on the outcome of an actor's behavior toward a recipient. These studies focused on interactions in which two agents are connected by an external goal (i.e., object-mediated social interaction) and revealed that the intent behind an action has a privileged role in evaluating the valence of a social interaction. The current study investigated whether the intent behind an action influences evaluation of contingent social interactions wherein one agent responds to another without referring to a specific target. To clarify this, we operationalized intent as harmful or harmless when one agent hit another (i.e., recipient), and manipulated the action's outcome by determining to what extent it changed the recipient's state (i.e., falling down or moving slightly). Results showed that in contingent interactions with both direct launching (i.e., the actor directly caused the change) and extended launching (i.e., the actor caused the change through a mediated block), when the action significantly affected the recipient, the agents were evaluated as having a more negative social interaction than when the influence was small; this effect was independent of the intent behind the action. Such findings demonstrated that evaluations of contingent social interactions are primarily influenced by an actor's causal role in the outcome, not the intent behind an action. This null effect of intent when evaluating social interaction contrasts with findings on object-mediated social interaction, which is consistent with human social evaluations relying on two dissociable systems: causal and intentional components.

## Introduction

When walking down the street, if you saw a person (i.e., actor) hit another person (i.e., recipient), how would you evaluate that social interaction? At first, you may rely on the recipient's response to the action, such as if the recipient fell down or moved slightly. The more harm the actor caused, the more negatively you may evaluate the social interaction. Alternatively, you may consider the actor's intentions. For example, the direction of the actor's face could provide hints about his or her intentions regarding the recipient (Baldwin and Baird, 2001; Calder et al., 2002; Frischen et al., 2007).

Our actions affect not only ourselves, but often influence others' actions or states of mind through social interactions, which are considered a building block of human society (Knoblich and Sebanz, 2008). Interestingly, brain connectivity during social interaction reflects individuals' social network structure (Schmälzle et al., 2017). The valence attributed to a social interaction (i.e., the extent to which the observed action is positive or negative) influences moral judgments and individual reputations (Hamlin et al., 2007; Ohtsuki et al., 2015). However, conclusions we reach when observing these interactions are strongly bound to the context in which an action occurs (Ullman et al., 2009; Csibra, 2017). Thus, further exploration is required to identify what information we rely on when we observe the actions of others, and how that information is used to assess the valence of social interactions.

In essence, social interactions are characterized as “A does X to B” or “A does X to B, and B responds with Y” (Hinde, 1976), which can result in the modification of each person's behavior (Gillin and Gillin, 1942). From such an operational view, the outcome of any action and its effect on others is an important cue when evaluating the valence of a social interaction. Furthermore, understanding an observed action relies on one's knowledge of the causal role between an action and its outcome (Dennett, 1989; Marien et al., 2015). Specifically, the effect of an action on the recipient determines the polarity of valence; a positive valence is attributed to a helpful outcome, while a negative valence is attributed to a harmful outcome. Additionally, how strongly the social interaction influences an agent determines the absolute value of its valence. For example, Hamlin et al. (2007) performed a study with 6- and 10-month-olds, and reported that the infants took into account how someone's actions affected someone else when determining the valence of a social interaction and, accordingly, this guided the infant's preferences regarding the individuals involved in that social interaction. Specifically, if the state change experienced by the recipient was caused by the actor, the infants could discriminate between social interactions with harmful outcomes or helpful outcomes, and showed preferences for helpful individuals over those who hindered others. Tatone et al. (2015) found that infants were able to distinguish between actions in which one person either benefits from the behavior of another or loses an object due to the other person's causal role, even if, on the surface, the two actions were similar.

Additionally, empirical studies have shown that adults interpret actions with stronger positive/negative effects on the recipient as more positive than actions with weaker positive/negative effects (Wu et al., 2018). That is, interactions that have more helpful/harmful outcomes are judged as more positive/negative than those that are less helpful/harmful. Research on moral judgment—a cognitive process that relies on the valence of a social interaction (Rai and Fiske, 2011; Gray et al., 2012)—supports this assertion (Lane and Anderson, 1976; Young et al., 2007; Cushman, 2008, 2015; Cushman et al., 2013; Baez et al., 2017), showing that participants judged actions resulting in negative outcomes as more morally wrong than actions resulting in neutral outcomes. Moreover, another study found that terrorists' moral judgements were mainly guided by outcomes rather than by intent (Baez et al., 2017). Hence, evaluations of social interactions rely heavily on how people's actions affect others.

When an individual interacts with someone else, he or she has underlying intentions driving his or her actions, which can be either harmful or harmless. Beyond an action's outcome on one's surroundings, one's underlying intentions are typically considered when actions are interpreted (Baldwin and Baird, 2001; Malle, 2004; Ames and Fiske, 2013, 2015). Thus, knowledge of both the intent and the outcome are crucial when judging an observable action. In other words, when an individual assesses the valence of a social interaction, both outcome and intent seem to contribute to individual evaluations of a given interaction.

Nevertheless, determining the role of intent when evaluating social interactions is not as clear as determining an action's outcome. Buon et al. (2013) claimed that people's evaluations of social interactions rely on two dissociable components: the causal roles of the agents and the content of the agents' intentions. This idea is consistent with the cognitive mechanism of moral judgement proposed by Cushman (2008), which posits that an assigned punishment is mainly dependent on the outcome caused by the transgressor, and the degree of moral wrongness attributed to the transgressor is heavily influenced by his or her intent. Further, Buon et al. (2013) suggested that the causal component is more intuitive and consumes fewer cognitive resources than the intentional component. In children, early outcome-based moral judgments are later (ages 4–8) replaced by intention-based moral judgments (Cushman et al., 2013). Further, in a fairness decision task (i.e., the ultimatum game), younger participants rejected unequal outcomes much more frequently than university students and weighed outcomes more than intent (Sutter, 2007). Hence, whether intent plays a role in social evaluations largely depends on the task and one's available cognitive resources.

Prior research indicates that we take into account the role of intent when evaluating social interactions (Sutter, 2007; Hamlin, 2013; Choi and Luo, 2015; Wu et al., 2018). For instance, Choi and Luo (2015) examined how 13-month-olds make sense of social interactions through infants' emergent theory-of-mind understanding. They found that if B accidentally hit C when A was present, infants seemed to accept that A could interact or not interact with B, but if B intentionally hit C, infants expected A to change his or her behavior and ignore B, suggesting that infants take intent into account when evaluating social interactions. Hamlin (2013) revealed that 8-month-olds preferred a puppet who failed to help over a puppet who failed to hinder, showing that infants considered the puppet's intent during subsequent social evaluations. These findings are in line with the results of other studies on moral judgment and social interaction (Young et al., 2007; Levine and Schweitzer, 2014; Nobes et al., 2016). In one study using adult participants, Levine and Schweitzer (2014) explored how intent and outcome jointly contributed to judgments regarding the moral character of an individual who lied, and found that people often perceive individuals who tell prosocial lies (i.e., lies told with the intention of benefiting the addressee) as more moral than those who tell harmful truths (i.e., truths that negatively affect the addressee). Young et al. (2007) found that attempted harm was judged more harshly than accidental harm and used functional magnetic resonance imaging to provide neural evidence that moral judgment was influenced by both intent and outcome.

A recent study provided more direct evidence of the role of intent in evaluation of the valence of a social interaction. Wu et al. (2018) used visual animations to operationalize an individual's actions as intentionally or accidentally influencing another and to manipulate the effect of the outcome on the affected agent to be either small (i.e., the actor's help/harm did not change whether the recipient could reach an apple) or great (i.e., due to the actor's help/harm, the recipient obtained or lost an apple), in both helpful and harmful contexts. They found that only when the actor intentionally affected the recipient did the outcome influence the evaluation of the social interaction. If the actor intentionally affected the recipient, the social interaction was perceived as more positive/negative when the help/harm had a significant effect compared to when the help/harm was minor; however, the strength of the help/harm did not affect an observer's assessment of the social interaction's valence when the effect was unintentional. Such findings indicate that the intent behind an actor's influence affects our evaluations of the valence of a social interaction. Nevertheless, the social interaction in this study was a specific kind, in which the actor modified the state of a recipient's external target (i.e., object-mediated social interaction), and the recipient's action reward changed accordingly. In short, the recipient's response to the actor resulted from the changed state of the directed object. However, two agents are often connected without external targets, but the tangible influence between them is manifested when one forms a contingent responsivity (i.e., synchronized responses) to the other (Gergely, 2010), such as when one agent knocks another over. This is also referred to as turn-taking (Bigelow and Rochat, 2006; Di Paolo et al., 2008; Marsh et al., 2009; Levinson, 2016). In such contingent interactions, it is unclear whether intent is taken into account when evaluating the valence of a social interaction.

In object-mediated social interactions, whether the incurred outcome on the recipient by the actor is positive or negative depends on how the recipient constructs the value of outcome in the mind (Ullman et al., 2009; Jara-Ettinger et al., 2016). For example, the increased costs on the recipient to obtain the object would mean the hindering, while the increased costs on the recipient to avoid the object may mean the helping. In this case, the perceived valence of this type of social interaction may appeal to the underlying intent, to obtain the value information of the changed object in terms of the recipient. In contrast to object-mediated social interactions, without appealing to the intent so much, contingent interactions are based on the action pattern of contingent responsivity and the observable effect (e.g., harm) of an actor on the recipient (Yin et al., 2018; Tauzin and Gergely, 2019). The nature of these interactions seems to imply that the key feature used to determine evaluations of them is whether contingent responsivity manifests in the outcome, with intent playing little to no role (Knoblich and Sebanz, 2008). Hence, we speculated that an individual's intentions would not modulate evaluations of social interactions or the effect of the outcome on such evaluations in a contingent response context. However, there is little evidence available in related literature to support this, so we remained open to other explanations.

Computer animation has begun to be used as an experimental tool in the study of social cognition. Inspired by this, we created a computer-animated contingent interaction in which one agent (actor) hits another agent (recipient), either via direct contact (i.e., direct launching; Experiment 1) or via an inanimate block (i.e., extended launching; Experiment 2), to simulate the action of bumping into another person. In this setting, the intent of the actor toward the recipient was manipulated by means of the direction of the agent's face (Baldwin and Baird, 2001; Calder et al., 2002; Frischen et al., 2007). Specifically, if the actor faced the recipient, the underlying intent to hit the recipient was seen as intentional harm; otherwise, it was seen as unintentional, as he or she could not see the other agent (Casallas et al., 2014). The study of intent and outcome in contingent social interactions can also be implemented by recording actions of real humans, as described in the first paragraph. However, if real humans are asked to emulate this event, when the person acted as the recipient knows the intentions of the person acted as the actor, it is difficult for the recipient to maintain the same action outcome caused by the actor, such as body gestures and facial expressions, in different intentions, as his or her actions could be slightly different due to automatic disturbances elicited by those intentions (Baldwin and Baird, 2001; Hudson et al., 2016; Quesque et al., 2016). In this case, the outcome of the recipient would be hard to pair with different intentions. As to simulating human actions with computer-animated agents, this method can strictly control for confounding factors, and has largely been documented to induce the perception of animacy, agency, intent, social interaction, etc., even at the very beginning of our development (Castelli et al., 2000; Csibra et al., 2003; Hamlin et al., 2007; Joyal et al., 2018). Though the perceived social information for computer-animated agents is reduced (Gobbini et al., 2011), if compared to actions recorded from real humans, it never disappears, and social evaluations for the computer-animated stimuli (e.g., moral judgement) show almost the same patterns as the human-generated stimuli (Chaminade et al., 2007; Mar et al., 2007; Hamlin, 2013).

Thus, the present study used computer-animated agents as stimuli. Further, we manipulated the intent of the actor as being either harmless (i.e., accidental hit) or harmful (i.e., intentional hit) toward the recipient by changing the direction of the actor's face. We also manipulated the outcome of the action on the recipient by determining to what extent the hit changed the recipient's state. To represent a small effect, the recipient moved slightly, and to represent a larger effect, the recipient fell down.

## Experiment 1: Direct Launching

This experiment was to investigate how an actor's intent and the outcome of his or her action's effects on the recipient influence evaluations of a contingent interaction's valence in direct launching events. In our simulation, we changed the perceived intentions of the actor toward the recipient by changing whether he faced the recipient or stood with his back to the recipient.

### Methods

#### Participants

A total of 44 paid adult volunteers (21 females; mean age = 22 years, ranging from 18 to 26) participated in this experiment. The sample size was determined via a power analysis based on a predicted effect using G^*^power 3 (Faul et al., 2009). Based on the results of our previous studies (Wu et al., 2018) with a medium effect size, we used a more conservative estimate, between a small and medium effect size (medium size: *f* = 0.25, and small size: *f* = 0.10, according to Cohen, 1988). With the alpha set at 0.05 and the power set at 0.80, the suggested sample size was 44 individuals. To maintain consistency in the number of participants between the two experiments, we used 44 valid participants in both. All participants had normal or corrected-to-normal vision and no reported history of neurological disorders. The study was reviewed and approved by the Research Ethics Board of the Department of Psychology at Ningbo University and was performed in accordance with the relevant guidelines and regulations. Participants provided written informed consent after learning the purpose and procedures of the experiment.

#### Apparatus and Stimuli

All stimuli were presented on a black background on a 19-inch CRT monitor (resolution = 800 × 600 pixels; refresh rate = 100 Hz) at a 60-cm viewing distance. The visual stimuli were created using Blender, a free, open-source 3D creation suite (https://www.blender.org/) and consisted of 3-s computer-animated events displayed within the full area of the screen (22.2° × 16.6°; see the Supplementary Videos for details). The events were presented in full at a 24.2° viewing distance using a 3D perspective view of 57°.

Each event involved two computer-animated agents of different colors and shapes, namely, a purple hexagon and a yellow square. Both included facial features (i.e., two eyes, a nose, and a mouth). In the simulation, the purple hexagon represented the actor. This figure was initially positioned in the left center of the screen, and a green ball was placed to its left. The green ball and the purple hexagon both moved, a situation particularly apparent when the purple hexagon faced the ball. In this case, it appeared that the purple hexagon avoided the green ball when the ball approached the hexagon. The purple hexagon moved toward the stationary yellow square (i.e., recipient) until they were adjacent; then, the purple hexagon stopped moving. Meanwhile, the yellow square started to move, and the two figures collided.

In this experiment, there were two possible outcomes when the two agents collided: (1) the yellow square moved slightly to the right due to the collision (i.e., small effect condition), similar to when a person jostles another and the latter stumbles slightly; or (2) the yellow square moved significantly to the right and appeared to fall down due to the collision (i.e., great effect condition), similar to when one person knocks another over. When the purple hexagon approached the yellow square, the actor's state was operationalized in one of the following two ways: (1) he faced the green ball to avoid its approach and appeared unable to see the yellow square, which was treated as a harmless intention; or (2) he faced the yellow square to approach it and was able to see the yellow square clearly, which was treated as a harmful intention. Taken together, this experiment involved a total of four conditions arranged in a 2 × 2 within-subject design, namely, intent (harmless intention vs. harmful intention) by outcome (small effect vs. great effect).

#### Procedure and Design

The four events were presented in random sequential order. Each event was replayed until participants pressed the spacebar to proceed to the next event. To overcome the possible memory load for the events (i.e., have to memorize the event contents during rating), participants were asked to answer the following three questions on a piece of paper as they watched the events (in this case, they could watch the videos anytime while answering, if necessary): “Do you think that the purple hexagon intentionally affected the yellow square?” (rated from 1 = completely disagree to 7 = completely agree); “Do you think that the changes to the yellow square were caused by the purple hexagon?” (rated from 1 = completely disagree to 7 = completely agree); and “What is the nature of the social interaction between the purple hexagon and the yellow square?” (rated from −5 = strongly negative/strongly hostile, to 5 = strongly positive/strongly friendly; 0 = a neutral social interaction). The first question was used as a manipulation check for participants' perception of the purple hexagon's intent (note that the word “influence” instead of “hit” was used in this question to maintain consistency between the two experiments). The second question sought to examine whether participants thought the effect on the yellow square was caused by the purple hexagon, while ruling out differences in evaluations of the valence of the social interaction across conditions due to different causality judgments. The third question aimed to determine participants' evaluations of the valence of the social interaction between the two agents.

To analyze the data, we used a traditional null hypothesis significance testing (NHST) procedure in the form of a repeated two-way analysis of variance (ANOVA) with intent and outcome as the two independent variables. However, in consideration of the drawbacks of NHST, such as p-hacking, we ran a Bayesian analysis (Cumming, 2014), which computes the ratio of the likelihood probability of two competing hypotheses. We computed the Bayes Factor (*BF*, H1/H0 as computed here) using JASP software (JASP Team, 2018). In Bayesian analysis, *BF* < 0.33 suggests substantial evidence against between-condition differences, while *BF* > 3.00 suggests substantial evidence supporting between-condition differences. Values between 0.33 and 3.00 are considered inconclusive (Dienes, 2014). For the raw data, please see the Supplementary Table 1.

### Results and Discussion

#### Intent

**Figure 1:**
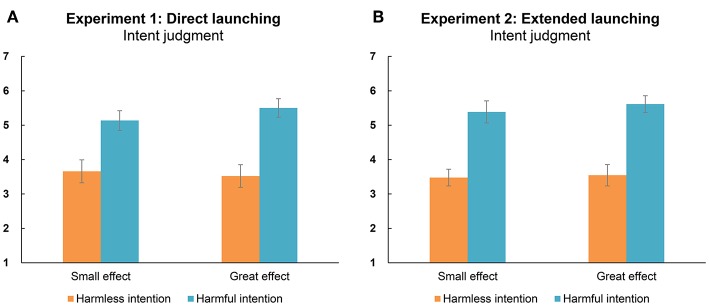
Judgment of the actor's intent (i.e., purple hexagon) affecting the recipient (i.e., yellow square) under different acting conditions in Experiment 1 **(A)** and Experiment 2 **(B)**. A score of 1 means “completely disagree” and 7 means “completely agree.” Error bars indicate the standard errors (±SE).

The overall results for participants' perceptions of the intent behind the purple hexagon's behavior are shown in Figure 1A. A two-way ANOVA with intent and outcome as factors revealed a significant main effect of intent [*F*_(1, 43)_ = 22.69, *p* < 0.001, $\eta_{p}^{2}$ = 0.35, *BF* = 5.80 × 10^7^]; neither the main effect of outcome [*F*_(1, 43)_ = 0.56, *p* = 0.457, $\eta_{p}^{2}$ = 0.01, *BF* = 0.18] nor the outcome × intent interaction [*F*_(1, 43)_ = 1.87, *p* = 0.179, $\eta_{p}^{2}$ = 0.04, *BF* = 0.35] was significant. These results indicated that participants perceived the hexagon's actions as intentionally causing an effect on the square in the harmful intention condition (*M* = 5.50; *SE* = 0.22), when compared to the harmless intention condition (*M* = 3.51; *SE* = 0.28). Furthermore, this effect was not modulated by the outcome. Therefore, participants could detect harmful intentions when the hexagon faced the square and harmless intentions when the hexagon did not face the square.

#### Causality

**Figure 2:**
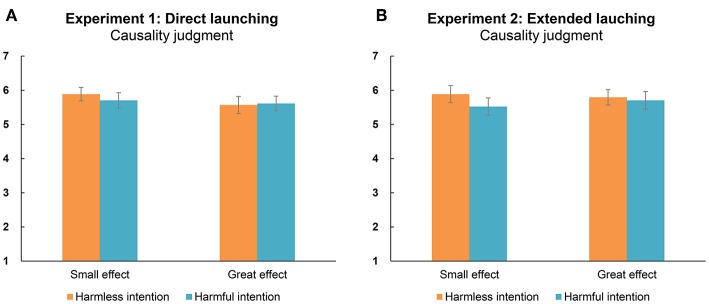
Causality judgment of the actor (i.e., purple hexagon) causing a change in the recipient's state (i.e., yellow square) under different acting conditions in Experiment 1 **(A)** and Experiment 2 **(B)**. A score of 1 means “completely disagree” and 7 means “completely agree.” Error bars indicate the standard errors (±SE).

Figure 2A shows the overall results of causality judgments. In all conditions, the scores of causality judgments were more than the median score on the provided seven-point scale [i.e., 4; *t*s_(43)_ > 6.20, *p*s < 0.001, *d*s > 0.93, *BF*s > 8.20 × 10^5^], suggesting that participants treated the effect on the yellow square as being caused by the purple hexagon. However, the ANOVA showed that none of the observed effects were significant [intent: *F*_(1, 43)_ = 0.09, *p* = 0.760, $\eta_{p}^{2}$ < 0.01, *BF* = 0.17; outcome: *F*_(1, 43)_ = 1.53, *p* = 0.222, $\eta_{p}^{2}$ = 0.03, *BF* = 0.27; interaction effect: *F*_(1, 43)_ = 0.37, *p* = 0.546, $\eta_{p}^{2}$ = 0.01, *BF* = 0.26]. Hence, the observed differences in the judgment of intent and the evaluation of the valence of the social interaction cannot be attributed to the causality judgment.

#### Valence of the Social Interaction

**Figure 3:**
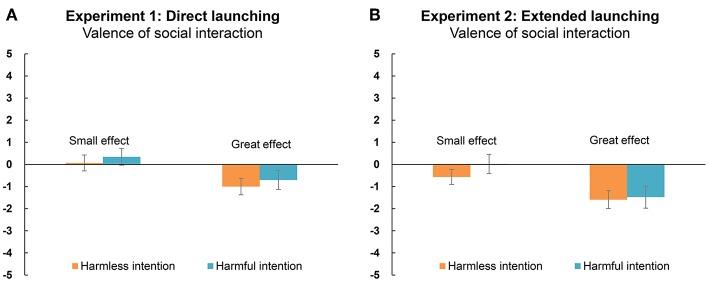
Evaluations of the valence of a social interaction between the actor (i.e., purple hexagon) and the recipient (i.e., yellow square) under different acting conditions in Experiment 1 **(A)** and Experiment 2 **(B)**. A score of −5 means a “strongly negative interaction,” 5 means “strongly positive interaction,” and 0 means “neural social interaction.” Error bars indicate the standard errors (±SE).

Figure 3A shows the overall results of participants' evaluations of the valence of the social interaction. The ANOVA revealed that only the main effect of outcome was significant [*F*_(1, 43)_ = 6.77, *p* = 0.013, $\eta_{p}^{2}$ = 0.14, *BF* = 12.86], suggesting that when the actor caused a significant effect on the recipient (*M* = −0.27; *SE* = 0.32), participants evaluated the social interaction between them as being more negative than when the actor caused a small effect on the recipient (*M* = −1.53; *SE* = 0.39). Meanwhile, neither the main effect of intent [*F*_(1, 43)_ = 0.52, *p* = 0.475, $\eta_{p}^{2}$ = 0.01, *BF* = 0.22] nor the interaction effect were significant [*F*_(1, 43)_ < 0.01, *p* = 0.956, $\eta_{p}^{2}$ < 0.01, *BF* = 0.22].

### Replication

In the above experiment, the actor was always the purple hexagon and the recipient was always the yellow square. This may have limited our findings by inducing preferences for one agent over the other. To determine if we could replicate the results from Experiment 1, we reversed the roles of the two agents and followed almost the same procedure as before, except that before watching the simulations, three new questions were asked as two static agents were presented on the screen: “How capable of planning actions do you think the purple hexagon is?” (rated from 1 = not at all to 7 = very much); “How capable of planning actions do you think the yellow square is?” (rated from 1 = not at all to 7 = very much); “Which one do you prefer between the purple hexagon and yellow square presented on the screen?” (1 = purple hexagon, 2 = yellow square, 3 = no preference). The first two questions were added to provide information about the agents' perceived agency, and the last one was added to investigate participants' preferences for the two agents. We recorded how long participants spent completing the questions regarding intent, causality, and valence of social interactions while watching the simulations (i.e., “rating time”). The rating time was recorded from the onset of replaying the simulations to the completion of the questions. As the rating time reflects the overall processing duration for understanding events and answering three different questions, we treated it cautiously when discussing how the rating time is related to evaluate social interactions. A different sample set of 44 paid adult volunteers (24 females; mean age = 22 years, ranging from 19 to 27 years) participated in this experiment.

#### Agency and Preference

Participants evaluated both agents' agency as a mean value of more than 3 [around the median score on the provided seven-point scale; purple hexagon: *M* = 3.77; *SE* = 0.22, *t*_(43)_ = 3.79, *p* < 0.001, *d* = 0.54, *BF* = 34.43; yellow square: *M* = 3.57; *SE* = 0.23, *t*_(43)_ = 2.51, *p* = 0.016, *d* = 0.38, *BF* = 2.66]. Furthermore, there was no significant difference in agency ratings between the two agents [*t*_(43)_ = 0.99, *p* = 0.329, *d* = 0.15, *BF* = 0.26]. Regarding preference, 14 out of 44 participants preferred the purple hexagon, 18 out of 44 participants preferred the yellow square, and 12 out of them selected the option of no preference between the two agents. A chi-square test revealed that participants did not have a differential preference for either of the two agents [$\chi_{\left(2\right)}^{2}$ = 1.27, *p* = 0.529].

#### Intent

The overall results for participants' perceptions of the intent behind the purple hexagon's behavior are shown in Table 1. A two-way ANOVA with intent and outcome as factors revealed that our manipulation for intent was valid. Specifically, there was a significant main effect of intent [*F*_(1, 43)_ = 62.23 *p* < 0.001, $\eta_{p}^{2}$ = 0.59, *BF* = 3.72 × 10^16^]. Neither the main effect of outcome [*F*_(1, 43)_ = 1.52, *p* = 0.225, $\eta_{p}^{2}$ = 0.03, *BF* = 0.20] nor the outcome × intent interaction [*F*_(1, 43)_ = 0.01, *p* = 0.910, $\eta_{p}^{2}$ < 0.01, *BF* = 0.21] was significant. These results indicated that participants did perceive the effects of the agent's actions to be more intentional in the harmful intention condition (*M* = 5.21; *SE* = 0.24) than in the harmless intention condition (*M* = 2.77; *SE* = 0.24).

**Table 1:** Descriptive results of different rating dimensions in replicating Experiment 1 (*M* ±*SE*).

	**Harmless intention**		**Harmful intention**	
	**Small effect**	**Great effect**	**Small effect**	**Great effect**
Intent	2.66 (0.27)	2.89 (0.27)	5.11 (0.24)	5.30 (0.29)
Causality	5.36 (0.29)	5.57 (0.24)	5.48 (0.23)	5.72 (0.20)
Valence of the social interaction	−0.43 (0.27)	−1.93 (0.34)	−0.16 (0.38)	−2.02 (0.42)
Rating time (s)	37.79 (2.55)	30.81 (2.48)	29.63 (2.50)	32.40 (2.38)

#### Causality

Table 1 shows the overall results of causality judgments. In all conditions, the scores of causality judgments were more than the median score on the provided seven-point scale [i.e., 4; *t*s_(43)_ > 4.68, *p*s < 0.001, *d*s > 0.70, *BF*s > 760.54], suggesting that participants treated the effect on the purple hexagon as being caused by the yellow square. However, the ANOVA showed that none of the observed effects were significant [intent: *F*_(1, 43)_ = 0.49, *p* = 0.486, $\eta_{p}^{2}$ = 0.01, *BF* = 0.20; outcome: *F*_(1, 43)_ = 0.97, *p* = 0.357, $\eta_{p}^{2}$ = 0.02, *BF* = 0.30; interaction effect: *F*_(1, 43)_ = 0.02, *p* = 0.884, $\eta_{p}^{2}$ < 0.01, *BF* = 0.21]. Hence, the observed differences in the judgment of intent and the evaluation of the valence of the social interaction cannot be attributed to the causality judgment.

#### Valence of the Social Interaction

Table 1 shows the overall results of participants' evaluations of the valence of the social interaction. The ANOVA revealed that only the main effect of outcome was significant [*F*_(1, 43)_ = 21.02, *p* < 0.001, $\eta_{p}^{2}$ = 0.33, *BF* = 48320.52], suggesting that when the actor caused a significant effect on the recipient (*M* = −1.98; *SE* = 0.32), participants evaluated the social interaction between them as being more negative than when the actor caused a small effect on the recipient (*M* = −0.30; *SE* = 0.26). Neither the main effect of intent [*F*_(1, 43)_ = 0.07, *p* = 0.791, $\eta_{p}^{2}$ < 0.01, *BF* = 0.26] nor the interaction effect was significant [*F*_(1, 43)_ = 0.67, *p* = 0.418, $\eta_{p}^{2}$ = 0.02, *BF* = 0.17].

#### Rating Time

**Figure 4:**
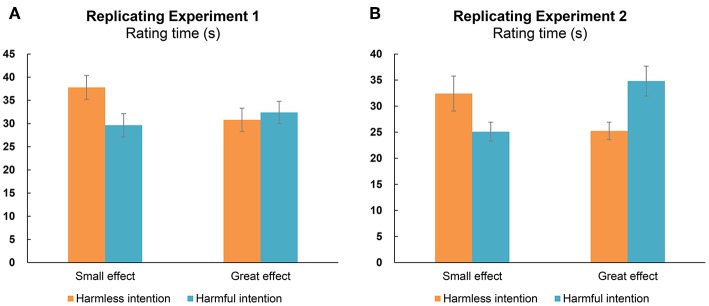
Rating times about completing the questions regarding intent, causality, and valence of social interactions under different acting conditions, in replicating Experiment 1 **(A)** and Experiment 2 **(B)**. Error bars indicate the standard errors (±SE).

Table 1 and Figure 4A show the rating times for different conditions. The ANOVA revealed that neither the main effect of intent [*F*_(1, 43)_ = 1.81, *p* = 0.185, $\eta_{p}^{2}$ = 0.04, *BF* = 0.53] nor the main effect of outcome [*F*_(1, 43)_ = 1.68, *p* = 0.202, $\eta_{p}^{2}$ = 0.04, *BF* = 0.27] was significant; however, the interaction effect between the two factors [*F*_(1, 43)_ = 7.51, *p* = 0.009, $\eta_{p}^{2}$ = 0.15, *BF* = 3.38] was significant. *Post-hoc* analysis of the simple effects revealed that when the actor caused a small effect on the recipient, participants judging the harmless intention condition needed more time to complete the three questions than for the harmful intention condition [*t*_(43)_ = 2.79, *p* = 0.008, *d* = 0.42, *BF* = 4.90]; however, this difference was not present when the actor caused a great effect on the recipient [*t*_(43)_ = 0.51, *p* = 0.614, *d* = 0.08, *BF* = 0.18].

Thus, participants did perceive the two figures as having agency, instead of as simply inanimate objects, and showed no differential preference for either of them. Importantly, the results of Experiment 1 were replicated.

## Experiment 2: Extended Launching

In Experiment 2, we tested the generalizability of the results of Experiment 1 and the robustness of the null effect of an actor's intent on social interaction evaluations. In general, in a contingent interaction, not only does an agent directly cause another's response, but there is often a causal chain. In this example, the second agent's contingent responsivity occurs when the first agent hits a mediator and this mediator works on the second agent, who responds in a specific manner (i.e., extended launching). For example, person A hits a table, the table moves and bumps into person B, who then falls down. Thus, in Experiment 2, we aimed to expand on the findings of Experiment 1, examining what would happen if the actor's effect on the recipient was extended by an inanimate object (e.g., a block).

### Methods

A different sample set of 44 paid adult volunteers (18 females; mean age = 23 years, ranging from 20 to 25 years) participated in the second experiment, in which a tan block was placed between the purple hexagon and yellow square. Except for this, both the stimuli and procedures were the same as in Experiment 1. In this case, the effect caused by the purple hexagon on the yellow square was extended by the block.

### Results and Discussion

#### Intent

The overall results regarding participants' evaluations of the purple hexagon's intent are shown in Figure 1B. The ANOVA showed that only intent yielded a significant main effect [*F*_(1, 43)_ = 81.05, *p* < 0.001, $\eta_{p}^{2}$ = 0.51, *BF* = 2.07 × 10^12^]. The results were consistent with our expectation that the harmful intention condition (*M* = 5.32; *SE* = 0.25) would result in higher ratings of intent relative to the harmless intention condition (*M* = 3.59; *SE* = 0.31). Neither the main effect of outcome [*F*_(1, 43)_ = 0.73, *p* = 0.399, $\eta_{p}^{2}$ = 0.02, *BF* = 0.18] nor the interaction effect [*F*_(1, 43)_ = 0.19, *p* = 0.666, $\eta_{p}^{2}$ < 0.01, *BF* = 0.24] was significant.

#### Causality

Figure 2B shows the overall results of causality judgments. Similar to the results of Experiment 1, participants scored causality higher than the median of the provided seven-point scale [i.e., 4; *t*s_(43)_ > 5.97, *p*s < 0.001, *d*s > 0.90, *BF*s > 3.94 × 10^4^], suggesting that they agreed the effect on the yellow square was caused by the purple hexagon. The ANOVA revealed that none of the observed effects were significant [intent: *F*_(1, 43)_ = 1.51, *p* = 0.226, $\eta_{p}^{2}$ < 0.03, *BF* = 0.42; outcome: *F*_(1, 43)_ = 0.13, *p* = 0.724, $\eta_{p}^{2}$ < 0.01, *BF* = 0.17; interaction effect: *F*_(1, 43)_ = 0.75, *p* = 0.393, $\eta_{p}^{2}$ = 0.02, *BF* = 0.33]. In this case, the demonstrated differences in the judgment of intent and the evaluation of the valence of the social interaction cannot be explained by the causality judgment.

#### Valence of the Social Interaction

The overall results for participants' evaluations of the valance of the social interaction between the two agents are shown in Figure 3B. We conducted an ANOVA with intent and outcome as factors for the evaluated valence of the social interaction. This showed that the main effect of outcome was significant [*F*_(1, 43)_ = 10.20, *p* = 0.003, $\eta_{p}^{2}$ = 0.19, *BF* = 58.81], suggesting that a significant effect on the recipient (*M* = −0.85; *SE* = 0.33) led to more negative evaluations of the social interaction than a small effect on the recipient (*M* = 0.21; *SE* = 0.30). Neither the main effect of intent [*F*_(1, 43)_ = 0.88, *p* = 0.354, $\eta_{p}^{2}$ = 0.02, *BF* = 0.25] nor the interaction effect [*F*_(1, 43)_ = 0.81, *p* = 0.374, $\eta_{p}^{2}$ = 0.02, *BF* = 0.259] was significant.

### Replication

Similar to Experiment 1, we conducted an experiment in which the roles of the two agents were reversed. A new sample set of 44 paid adult volunteers (26 females; mean age = 21 years, ranging from 18 to 25) participated in this experiment.

#### Agency and Preference

Participants evaluated both agents' agency as more than a mean value of 3 [around the median score on the provided seven-point scale; purple hexagon: *M* = 3.77; *SE* = 0.18, *t*_(43)_ = 4.35, *p* < 0.001, *d* = 0.66, *BF* = 282.96; yellow square: *M* = 3.64; *SE* = 0.17, *t*_(43)_ = 3.83, *p* < 0.001, *d* = 0.58, *BF* = 2.66]. Additionally, there was no significant difference in agency ratings between the two agents [*t*_(43)_ = 0.99, *p* = 0.329, *d* = 0.15, *BF* = 66.10]. Regarding preference, 14 out of 44 participants preferred the purple hexagon, 18 out of 44 participants preferred the yellow square, and 12 out of them selected the option of no preference between the two agents. A chi-square test revealed that participants did not have a differential preference for either of the two agents [$\chi_{\left(2\right)}^{2}$ = 0.86, *p* = 0.649].

#### Intent

The overall results for participants' perceptions of the intent behind the purple hexagon's behavior are shown in Table 2. A two-way ANOVA with intent and outcome as factors confirmed that our manipulation of intent was valid. Specifically, a significant main effect of intent was found [*F*_(1, 43)_ = 60.87, *p* < 0.001, $\eta_{p}^{2}$ = 0.59, *BF* = 1.28 × 10^21^]. Neither the main effect of outcome [*F*_(1, 43)_ = 0.82, *p* = 0.524, $\eta_{p}^{2}$ = 0.02, *BF* = 0.17] nor the outcome × intent interaction [*F*_(1, 43)_ = 0.41, *p* = 0.524, $\eta_{p}^{2}$ = 0.01, *BF* = 0.18] was significant. These results indicated that participants indeed perceived the actor's effect on the recipient as more intentional in the harmful intention condition (*M* = 5.42; *SE* = 0.22) when compared to the harmless intention condition (*M* = 2.88; *SE* = 0.27).

**Table 2:** Descriptive results of different rating dimensions in replicating Experiment 2 (*M* ±*SE*).

	**Harmless intention**		**Harmful intention**	
	**Small effect**	**Great effect**	**Small effect**	**Great effect**
Intent	2.80 (0.27)	2.95 (0.28)	5.41 (0.22)	5.43 (0.24)
Causality	5.75 (0.21)	5.89 (0.19)	5.89 (0.18)	5.70 (0.22)
Valence of social interaction	−0.11 (0.32)	−1.02 (0.31)	0.02 (0.44)	−1.27 (0.42)
Rating time (s)	32.41 (3.32)	25.25 (1.66)	25.11 (1.80)	34.81 (2.83)

#### Causality

Table 2 shows the overall results of causality judgments. In all conditions, the scores of causality judgments were more than the median score on of the provided seven-point scale [i.e., 4; *t*s_(43)_ > 7.52, *p*s < 0.001, *d*s > 1.13, *BF*s > 5.18 × 10^6^], suggesting that participants treated the effect on the yellow square as being caused by the purple hexagon. However, the ANOVA showed that none of the observed effects were significant [intent: *F*_(1, 43)_ = 0.03, *p* = 0.859, $\eta_{p}^{2}$ < 0.01, *BF* = 0.17; outcome: *F*_(1, 43)_ = 0.02, *p* = 0.903, $\eta_{p}^{2}$ < 0.01, *BF* = 0.16; interaction effect: *F*_(1, 43)_ = 2.05, *p* = 0.160, $\eta_{p}^{2}$ = 0.05, *BF* = 0.33]. Hence, the observed differences in the judgment of intent and the evaluation of the valence of the social interaction cannot be attributed to the causality judgment.

#### Valence of the Social Interaction

Table 2 shows the overall results of participants' evaluations of the valence of the social interaction. The ANOVA revealed that only the main effect of outcome was significant [*F*_(1, 43)_ = 33.42, *p* < 0.001, $\eta_{p}^{2}$ = 0.44, *BF* = 48320.52], suggesting that when the actor caused a significant effect to the recipient (*M* = −1.15; *SE* = 0.31), participants evaluated the social interaction between them as being more negative than when the actor caused a small effect to the recipient (*M* = −0.05; *SE* = 0.32). Meanwhile, neither the main effect of intent [*F*_(1, 43)_ = 0.02, *p* = 0.883, $\eta_{p}^{2}$ < 0.01, *BF* = 0.26] nor the interaction effect was significant [*F*_(1, 43)_ = 1.13, *p* = 0.294, $\eta_{p}^{2}$ = 0.03, *BF* = 0.17].

#### Rating Time

Table 2 and Figure 4B show the rating times for different conditions. The ANOVA revealed that neither the main effect of intent [*F*_(1, 43)_ = 0.17, *p* = 0.685, $\eta_{p}^{2}$ < 0.01, *BF* = 0.18] nor the main effect of outcome [*F*_(1, 43)_ = 0.32, *p* = 0.575, $\eta_{p}^{2}$ < 0.01, *BF* = 0.19] was significant; however, the interaction effect between the two factors [*F*_(1, 43)_ = 28.91, *p* < 0.001, $\eta_{p}^{2}$ = 0.40, *BF* = 121.27] was significant. *Post-hoc* analysis of the simple effects revealed that when the actor caused a small effect to the recipient, participants needed more time to complete the three questions in the harmless intention condition than in the harmful intention condition [*t*_(43)_ = 3.27, *p* = 0.002, *d* = 0.49, *BF* = 15.13], but this pattern was reversed when the actor caused a great effect on the recipient [*t*_(43)_ = 2.15, *p* = 0.037, *d* = 0.32, *BF* = 1.30]. When the actor caused a great effect on the recipient, although there was a significant difference between the harmless intention and harmful intention conditions, the evidence ratio (i.e., *BF* = 1.30) supporting this difference was inconclusive. Hence, these results regarding the rating times were almost consistent with Experiment 1.

Thus, the results of Experiment 2 were replicated.

## General Discussion

Evaluations of the valence of social interactions play an essential role in our daily lives. These evaluations rely heavily on the outcome of one person's actions toward another, and research has suggested that our perception of the intent behind these actions may affect our assessment of the overall situation (Hamlin, 2013; Wu et al., 2018). In contrast to previous studies that focused on an interaction in which two agents were connected by an external target (i.e., object-mediated social interaction; Wu et al., 2018), the current study investigated how the outcome of and intent behind an action influenced evaluations of contingent social interactions, wherein one agent responded to another without referring to a specific target. Results showed that only the outcome of an actor's behavior toward a recipient determined how a social interaction was evaluated. Specifically, in both contingent interaction contexts—direct launching and extended launching—when an action caused a significant effect on the recipient, the agents were evaluated as having a stronger social interaction than when the effect was small. This effect was independent from the intent behind the action.

Although it is risky to interpret the null effect, in both experiments, we found a consistent null effect of intent on evaluations of social interactions, even when the roles of the agents were switched. Beyond the values obtained from conducting the traditional NHST procedure, the computed BFs were <0.33 for all effects related to the intent factor, suggesting substantial evidence against a difference in the valence of social interaction involving intentionally hitting compared to accidentally hitting. Such a null effect cannot be explained by participants' inability to extract information regarding intent from computer animations, as in both experiments, participants perceived actions as more intended to affect the recipient in the harmful intention condition when compared to the harmless intention condition. Moreover, the null effect cannot be attributed to ignoring the intent of the computer-animated agents, since, in both experiments, when the actor unintentionally hit the recipient, participants needed more time to complete the three rating questions in the condition with a small effect on the recipient than in the condition with a large effect on the recipient, but this did not occur when the actor intentionally hit the recipient, showing that participants considered the agents to be capable of intent. In Experiment 2, we found the opposite effect; when the actor intentionally hit the recipient, participants needed more time to complete the three rating questions in the condition with a great effect on the recipient than in the condition with a small effect on the recipient. This evidence is inconclusive, but these findings may suggest that in addition to outcome, intent is also taken into account when evaluating social interactions. Furthermore, in a previous study conducted by Wu et al. (2018) using computer-animated agents to simulate object-mediated social interactions, intent was absolutely noted and had a privileged role in the evaluation of the valence of a social interaction. Hence, we suggest that the intent underlying an action's effect on a recipient plays no crucial role in evaluations of the valence of a social interaction, at least within the current context of computer-animated actions.

Our findings are in line with previous suggestions that the outcome of an individual's action toward another person determines observers' evaluations of the social interaction (Hamlin et al., 2007; Tatone et al., 2015; Tatone, 2017; Wu et al., 2018). Notably, the current study extended this conclusion to a new situation wherein a contingent interaction established a social link between individuals without direction toward an external target. However, in contrast to previous research (Wu et al., 2018), we found that an agent's intent to affect a recipient did not influence observers' evaluations of the social interaction. A critical difference between these studies is the interaction's form; we used contingent responsivity to link the two agents, whereas Wu et al. (2018) used an external target for this link. As suggested, evaluations of social interactions rely on two dissociable components, one of which is sensitivity to the causal role of agents and the other to the content of agents' intentions. The differing results may therefore imply that two distinct mechanisms are at work in evaluating these different forms of social interaction. Specifically, evaluations of contingent interactions mainly rely on causal structure, and evaluations of object-mediated social interactions may take intent into account.

Contingent interactions are believed to originate from early turn-taking experiences in mother/infant interactions and are demonstrated particularly profoundly in conversational structure (Bigelow and Rochat, 2006; Csibra, 2010). Most importantly, contingent responsivity is always dependent on the detection of causal relationships (O'Callaghan et al., 2018). Infants are likely unable to understand a parent's intent in conversations; thus, the caused effect (i.e., action outcome) in contingent responsivity is the key determinant for computing the valence of a social interaction, with intent having little to no importance. This early action template regarding a cause-based heuristic process might form the basis for inferences made regarding contingent interactions later in life as well.

When understanding object-mediated social interactions (i.e., two persons are connected with a specific external target), observers may rely on the balance of the expected benefits and incurred costs when interpreting the interaction, expecting that individuals' intentions or actions would be made to maximize their utilities (i.e., naïve utility calculus; Jara-Ettinger et al., 2016). Specifically, the costs of affecting others might have been repaid by the social interaction goal, as the second-order expected reward resulted in the intended outcomes (i.e., helping or hindering). Hence, when an intended outcome is observed (i.e., helpful intentions or harmful intentions), the perceived valence of the social interaction tends to be enhanced, thereby showing that intent influences the evaluation of social interactions. In such evaluations, the amount of benefits and costs is dependent on the recipient's mental state about how the incurred outcome on the others is expected and rewarded; hence, intent must be considered when evaluating the valence of a social interaction. However, this interpretation should be confirmed in subsequent research.

Buon et al. (2013) posited that, during social evaluations, the causal component is more automatic than the intentional component, and cognitive resources are consumed when integrating the two. Hence, if cognitive resources are limited, social evaluations are mainly based on the casual roles of the agents. This seems inconsistent with our study, in which participants had enough time and resource to complete tasks, while in the study conducted by Buon et al. (2013), participants compared two events with different causal roles or intentions at the same time, and this direct comparison may have led participants to focus on information regarding intent when cognitive resources were available. In our study, participants were required to report their evaluations without direct comparisons, and evaluating contingent interaction may only utilize the subsystem of detecting causal roles; however, in the study of Wu et al. (2018), the evaluation of the recipient's benefits and costs may have activated the intent subsystem. Preliminary results from a study in which deliberate thinking is manipulated before the evaluation of the valence of social interaction show that outcome and intention interact in affecting valence ratings^1^. This suggests that interpreting the role of intent when evaluating contingent social interactions is not automatic but depends on the magnitude of the outcome, consistent with the idea of two systems affecting the evaluation of social interactions.

One limitation of the present study is that actions were simulated using computer-generated animations. Although this method does provide solid theoretical evidence, actual social perception (e.g., inferences about goal pursuits) is more complex; therefore, drawing relatively broad conclusions about real-world social interactions is not possible. Further research should employ ecologically valid stimuli (e.g., videos depicting real people), which would allow for broader generalizations. Furthermore, during social evaluations, individuals may have already learned social information about the involved agents, such as reputation, personality, etc., through direct interaction or stored stereotypes, and previous research has suggested that this prior information heavily influences trustworthiness evaluations (Delgado et al., 2005; Fouragnan et al., 2013; Li et al., 2017; Ponsi et al., 2017). In future studies, it will be important to consider these factors as well.

## Conclusion

In this study, we demonstrated that in contingent social interactions, evaluations of the valence of a social interaction are primarily influenced by the outcome of an action taken toward someone else, not the intent behind this action. The null effect of intent contrasts with findings of previous studies on social interactions established via an external target. Further investigations are needed to address the possible mechanisms that may explain these disparate findings.

## Data Availability Statement

All datasets generated for this study are included in the article/Supplementary Material.

## Ethics Statement

The studies involving human participants were reviewed and approved by Research Ethics Board of the Department of Psychology at Ningbo University. The patients/participants provided their written informed consent to participate in this study.

## Author Contributions

JY conceived and designed the experiments. XH, YY, and XW performed the experiments and analyzed the data. JY and XH wrote the manuscript.

### Conflict of Interest

The authors declare that the research was conducted in the absence of any commercial or financial relationships that could be construed as a potential conflict of interest.

## Footnotes

^1^Specifically, the main effect of intent was not significant [*F*_(1, 49)_ = 0.18, *p* = 0.675, $\eta_{p}^{2}$ < 0.01]; the main effect of outcome [*F*_(1, 49)_ = 16.84, *p* < 0.001, $\eta_{p}^{2}$ = 0.26] was significant and the interaction effect [*F*_(1, 49)_ = 8.99, *p* = 0.004, $\eta_{p}^{2}$ = 0.16] also reached the level of significance. The analysis of simple effects showed that when the actor caused a small effect on the recipient, the social interaction with harmful intentions (i.e., intentional) tended to be evaluated more negatively than the social interaction with harmless intentions [i.e., unintentional; *t*_(49)_ = 1.92, *p* = 0.060, *d* = 0.27], but this difference was not present when the actor caused a great effect on the recipient [*t*_(43)_ = 1.24, *p* = 0.220, *d* = 0.18].

## References

[B1] Ames D. L. Fiske S. T. (2013). Intentional harms are worse, even when they're not. Psychol. Sci. 24, 1755–1762. 10.1177/0956797613480507 23878021 PMC4470288

[B2] Ames D. L. Fiske S. T. (2015). Perceived intent motivates people to magnify observed harms. Proc. Natl. Acad. Sci. U.S.A. 112, 3599–3605. 10.1073/pnas.1501592112 25733850 PMC4378403

[B3] Baez S. Herrera E. García A. M. Manes F. Young L. Ibáñez A. (2017). Outcome-oriented moral evaluation in terrorists. Nat. Hum. Behav. 1:0118 10.1038/s41562-017-0118

[B4] Baldwin D. A. Baird J. A. (2001). Discerning intentions in dynamic human action. Trends Cogn. Sci. 5, 171–178. 10.1016/S1364-6613(00)01615-6 11287271

[B5] Bigelow A. E. Rochat P. (2006). Two-month-old infants' sensitivity to social contingency in mother–infant and stranger–infant interaction. Infancy 9, 313–325. 10.1207/s15327078in0903_3 33412678

[B6] Buon M. Jacob P. Loissel E. Dupoux E. (2013). A non-mentalistic cause-based heuristic in human social evaluations. Cognition 126, 149–155. 10.1016/j.cognition.2012.09.006 23177140

[B7] Calder A. J. Lawrence A. D. Keane J. Scott S. K. Owen A. M. Christoffels I.. (2002). Reading the mind from eye gaze. Neuropsychologia 40, 1129–1138. 10.1016/S0028-3932(02)00008-8 11931917

[B8] Casallas J. S. Oliver J. H. Kelly J. W. Merienne F. Garbaya S. (2014). Using relative head and hand-target features to predict intention in 3D moving-target selection, in Virtual Reality (VR) (New York, NY: Curran Associates, Inc.), 51–56. 10.1109/VR.2014.6802050

[B9] Castelli F. Happé F. Frith U. Frith C. (2000). Movement and mind: a functional imaging study of perception and interpretation of complex intentional movement patterns. NeuroImage 12, 314–325. 10.1006/nimg.2000.0612 10944414

[B10] Chaminade T. Hodgins J. Kawato M. (2007). Anthropomorphism influences perception of computer-animated characters' actions. Soc. Cogn. Affect. Neurosci. 2, 206–216. 10.1093/scan/nsm017 18985142 PMC2569803

[B11] Choi Y. J. Luo Y. (2015). 13-month-olds' understanding of social interactions. Psychol. Sci. 26, 274–283. 10.1177/0956797614562452 25630442

[B12] Cohen J. (1988). Statistical Power Analysis for the Behavioral Sciences, 2nd Edn. Hillsdale, NJ: Lawrence Erlbaum Associates.

[B13] Csibra G. (2010). Recognizing communicative intentions in infancy. Mind Lang. 25, 141–168. 10.1111/j.1468-0017.2009.01384.x

[B14] Csibra G. (2017). Cognitive science: modelling theory of mind. Nat. Hum. Behav. 1:0066 10.1038/s41562-017-0066

[B15] Csibra G. Bíró S. Koós O. Gergely G. (2003). One year old infants use teleological representations of actions productively. Cogn. Sci. 27, 111–133. 10.1016/S0364-0213(02)00112-X

[B16] Cumming G. (2014). The new statistics: why and how. Psychol. Sci. 25, 7–29. 10.1177/0956797613504966 24220629

[B17] Cushman F. (2008). Crime and punishment: Distinguishing the roles of causal and intentional analyses in moral judgment. Cognition 108, 353–380. 10.1016/j.cognition.2008.03.006 18439575

[B18] Cushman F. (2015). Deconstructing intent to reconstruct morality. Curr. Opin. Psychol. 6, 97–103. 10.1016/j.copsyc.2015.06.003

[B19] Cushman F. Sheketoff R. Wharton S. Carey S. (2013). The development of intent-based moral judgment. Cognition 127, 6–21. 10.1016/j.cognition.2012.11.008 23318350

[B20] Delgado M. R. Frank R. H. Phelps E. A. (2005). Perceptions of moral character modulate the neural systems of reward during the trust game. Nat. Neurosci. 8, 1611–1618. 10.1038/nn1575 16222226

[B21] Dennett D. C. (1989). The intentional stance. Philos. Books 30, 169–172. 10.1111/j.1468-0149.1989.tb02170.x

[B22] Di Paolo E. A. Rohde M. Iizuka H. (2008). Sensitivity to social contingency or stability of interaction? Modelling the dynamics of perceptual crossing. New Ideas Psychol. 26, 278–294. 10.1016/j.newideapsych.2007.07.006

[B23] Dienes Z. (2014). Using Bayes to get the most out of non-significant results. Front. Psychol. 5:781. 10.3389/fpsyg.2014.00781 25120503 PMC4114196

[B24] Faul F. Erdfelder E. Buchner A. Lang A. G. (2009). Statistical power analyses using G^*^ Power 3.1: tests for correlation and regression analyses. Behav. Res. Methods 41, 1149–1160. 10.3758/BRM.41.4.1149 19897823

[B25] Fouragnan E. Chierchia G. Greiner S. Neveu R. Avesani P. Coricelli G. (2013). Reputational priors magnify striatal responses to violations of trust. J. Neurosci. 33, 3602–3611. 10.1523/JNEUROSCI.3086-12.2013 23426687 PMC6619519

[B26] Frischen A. Bayliss A. P. Tipper S. P. (2007). Gaze cueing of attention: visual attention, social cognition, and individual differences. Psychol. Bull. 133, 694–724. 10.1037/0033-2909.133.4.694 17592962 PMC1950440

[B27] Gergely G. (2010). Kinds of agents: the origins of understanding instrumental and communicative agency, in Blackwell Handbook of Childhood Cognitive Development, 2nd Edn. ed Goshwami U. (Oxford: Blackwell), 76–105. 10.1002/9781444325485.ch3

[B28] Gillin J. L. Gillin J. P. (1942). An Introduction to Sociology. New York, NY: Macmillan.

[B29] Gobbini M. I. Gentili C. Ricciardi E. Bellucci C. Salvini P. Laschi C.. (2011). Distinct neural systems involved in agency and animacy detection. J. Cogn. Neurosci. 23, 1911–1920. 10.1162/jocn.2010.21574 20849234

[B30] Gray K. Waytz A. Young L. (2012). The moral dyad: a fundamental template unifying moral judgment. Psychol. Inq. 23, 206–215. 10.1080/1047840X.2012.686247 22815620 PMC3396360

[B31] Hamlin J. K. (2013). Failed attempts to help and harm: Intention versus outcome in preverbal infants' social evaluations. Cognition 128, 451–474. 10.1016/j.cognition.2013.04.004 23811094

[B32] Hamlin J. K. Wynn K. Bloom P. (2007). Social evaluation by preverbal infants. Nature 450, 557–559. 10.1038/nature06288 18033298

[B33] Hinde R. A. (1976). Interactions, relationships and social structure. Man 11, 1–17. 10.2307/2800384

[B34] Hudson M. Nicholson T. Simpson W. A. Ellis R. Bach P. (2016). One step ahead: The perceived kinematics of others' actions are biased toward expected goals. J. Exp. Psychol. Gen. 145, 1–7. 10.1037/xge0000126 26595838 PMC4694084

[B35] Jara-Ettinger J. Gweon H. Schulz L. E. Tenenbaum J. B. (2016). The naïve utility calculus: Computational principles underlying commonsense psychology. Trends Cogn. Sci. 20, 589–604. 10.1016/j.tics.2016.05.011 27388875

[B36] JASP Team (2018). JASP (Version 0.9) [Computer Software]. Amsterdam.

[B37] Joyal C. C. Neveu S.-M. Boukhalfi T. Jackson P. L. Renaud P. (2018). Suppression of sensorimotor Alpha power associated with pain expressed by an avatar: a preliminary EEG study. Front. Hum. Neurosci. 12:273. 10.3389/fnhum.2018.00273 30038564 PMC6046452

[B38] Knoblich G. Sebanz N. (2008). Evolving intentions for social interaction: from entrainment to joint action. Philos. Trans. R. Soc. Lond. B. Biol. Sci. 363, 2021–2031. 10.1098/rstb.2008.0006 18292061 PMC2606699

[B39] Lane J. Anderson N. H. (1976). Integration of intention and outcome in moral judgment. Mem. Cognit. 4, 1–5. 10.3758/BF03213247 21286951

[B40] Levine E. E. Schweitzer M. E. (2014). Are liars ethical? On the tension between benevolence and honesty. J. Exp. Soc. Psychol. 53, 107–117. 10.1016/j.jesp.2014.03.005

[B41] Levinson S. C. (2016). Turn-taking in human communication–Origins and implications for language processing. Trends Cogn. Sci. 20, 6–14. 10.1016/j.tics.2015.10.010 26651245

[B42] Li D. Meng L. Ma Q. (2017). Who deserves my trust? Cue-elicited feedback negativity tracks reputation learning in repeated social interaction? Front. Hum. Neurosci. 11:307. 10.3389/fnhum.2017.00307 28663727 PMC5471337

[B43] Malle B. F. (2004). How the Mind Explains Behavior: Folk Explanations, Meaning, and Social Interaction. Cambridge, MA: MIT Press 10.7551/mitpress/3586.001.0001

[B44] Mar R. A. Kelley W. M. Heatherton T. F. Macrae C. N. (2007). Detecting agency from the biological motion of veridical vs animated agents. Soc. Cogn. Affect. Neurosci. 2, 199–205. 10.1093/scan/nsm011 18985141 PMC2569809

[B45] Marien H. Aarts H. Custers R. (2015). The interactive role of action-outcome learning and positive affective information in motivating human goal-directed behavior. Motivat. Sci. 1, 165–183. 10.1037/mot0000021

[B46] Marsh K. L. Richardson M. J. Schmidt R. C. (2009). Social connection through joint action and interpersonal coordination. Top. Cogn. Sci. 1, 320–339. 10.1111/j.1756-8765.2009.01022.x 25164936

[B47] Nobes G. Panagiotaki G. Bartholomew K. J. (2016). The influence of intention, outcome and question-wording on children's and adults' moral judgments. Cognition 157, 190–204. 10.1016/j.cognition.2016.08.019 27649094

[B48] O'Callaghan C. Vaghi M. M. Brummerloh B. Cardinal R. N. Robbins T. W. (2018). Impaired awareness of action-outcome contingency and causality during healthy ageing and following ventromedial prefrontal cortex lesions. Neuropsychologia 128, 282–289. 10.1016/j.neuropsychologia.2018.01.021 29355648 PMC6562272

[B49] Ohtsuki H. Iwasa Y. Nowak M. A. (2015). Reputation effects in public and private interactions. PLoS Comput. Biol. 11:e1004527. 10.1371/journal.pcbi.1004527 26606239 PMC4659694

[B50] Ponsi G. Panasiti M. S. Aglioti S. M. Liuzza M. T. (2017). Right-wing authoritarianism and stereotype-driven expectations interact in shaping intergroup trust in one-shot vs multiple-round social interactions. PLoS ONE 12:e0190142. 10.1371/journal.pone.0190142 29284019 PMC5746237

[B51] Quesque F. Delevoye-Turrell Y. Coello Y. (2016). Facilitation effect of observed motor deviants in a cooperative motor task: evidence for direct perception of social intention in action. Q. J. Exp. Psychol. 69, 1451–1463. 10.1080/17470218.2015.1083596 26288247

[B52] Rai T. S. Fiske A. P. (2011). Moral psychology is relationship regulation: moral motives for unity, hierarchy, equality, and proportionality. Psychol. Rev. 118, 57–75. 10.1037/a0021867 21244187

[B53] Schmälzle R. O'Donnell M. B. Garcia J. O. Cascio C. N. Bayer J. Bassett D. S.. (2017). Brain connectivity dynamics during social interaction reflect social network structure. Proc. Natl. Acad. Sci. U.S.A. 114, 5153–5158. 10.1073/pnas.1616130114 28465434 PMC5441802

[B54] Sutter M. (2007). Outcomes versus intentions: On the nature of fair behavior and its development with age. J. Econ. Psychol. 28, 69–78. 10.1016/j.joep.2006.09.001

[B55] Tatone D. (2017). The naïve sociology of resource transfer (Doctoral dissertation). Central European University, Budapest, Hungary.

[B56] Tatone D. Geraci A. Csibra G. (2015). Giving and taking: Representational building blocks of active resource-transfer events in human infants. Cognition 137, 47–62. 10.1016/j.cognition.2014.12.007 25614012 PMC4641319

[B57] Tauzin T. Gergely G. (2019). Variability of signal sequences in turn-taking exchanges induces agency attribution in 10.5-mo-olds. Proc. Natl. Acad. Sci. U.S.A. 116, 15441–15446. 10.1073/pnas.1816709116 31308230 PMC6681728

[B58] Ullman T. Baker C. Macindoe O. Evans O. Goodman N. Tenenbaum J. B. (2009). Help or hinder: Bayesian models of social goal inference, in Advances in Neural Information Processing Systems, Vol. 22, eds Bengio Y. Schuurmans D. Lafferty J. Williams C. K. I. Culotta A. (Vancouver, BC: NIPS Foundation), 1874–1882.

[B59] Wu X. Hua R. Yang Z. Yin J. (2018). The influence of intention and outcome on evaluations of social interaction. Acta. Psychol. 182, 75–81. 10.1016/j.actpsy.2017.11.010 29149691

[B60] Yin J. Chen M. Wang X. Ding X. (2018). Fleeing or not: responsivity of a chased target influences the cognitive representation of the chasing action. Atten. Percept. Psychophys. 80, 1205–1213. 10.3758/s13414-018-1508-9 29557036

[B61] Young L. Cushman F. Hauser M. Saxe R. (2007). The neural basis of the interaction between theory of mind and moral judgment. Proc. Natl. Acad. Sci. U.S.A. 104, 8235–8240. 10.1073/pnas.0701408104 17485679 PMC1895935

