# Supplementary material for: Outcome-Based Evaluations of Social Interaction Valence in a Contingent Response Context
Source: Front Psychol. 2019 Nov 20;10:2557. doi: 10.3389/fpsyg.2019.02557 (PMC6879419; doi:10.3389/fpsyg.2019.02557)
Supplement: Supplementary file 2 [file Table_2.DOCX]

**Supplementary Information**

Here, we provided totally 8 demos for two experiments. The size of each video frame is

800 × 600 pixels and the lasting time for each demo is 3 s with 50 frames per second. If you are interested in learning more about the demos and the experiments, please contact with the corresponding author: Jun Yin ([yinjun1@nbu.edu.cn](mailto:yinjun1@nbu.edu.cn)).

**Video 1:** The video of showing that the actor (i.e., purple hexagon) intentionally affects the recipient (i.e., yellow square), and exerts a great effect on the recipient in the context of direct launching in Experiment 1.

**Video 2:** The video of showing that the actor (i.e., purple hexagon) intentionally affects the recipient (i.e., yellow square), and exerts a small effect on the recipient in the context of direct launching in Experiment 1.

**Video 3:** The video of showing that the actor (i.e., purple hexagon) unintentionally affects the recipient (i.e., yellow square), but exerts a great effect on the recipient in the context of direct launching in Experiment 1.

**Video 4:** The video of showing that the actor (i.e., purple hexagon) unintentionally affects the recipient (i.e., yellow square), but exerts a small effect on the recipient in the context of direct launching in Experiment 1.

**Video 5:** The video of showing that the actor (i.e., purple hexagon) intentionally affects the recipient (i.e., yellow square), and exerts a great effect on the recipient in the context of extended launching in Experiment 2.

**Video 6:** The video of showing that the actor (i.e., purple hexagon) intentionally affects the recipient (i.e., yellow square), and exerts a small effect on the recipient in the context of extended launching in Experiment 2.

**Video 7:** The video of showing that the actor (i.e., purple hexagon) unintentionally affects the recipient (i.e., yellow square), but exerts a great effect on the recipient in the context of extended launching in Experiment 2.

**Video 8:** The video of showing that the actor (i.e., purple hexagon) unintentionally affects the recipient (i.e., yellow square), but exerts a small effect on the recipient in the context of extended launching in Experiment 2.
